# Relationship‐Building and Care Practices of Home‐Visiting Nurses Caring for Children With Medical Complexity and Their Families in Japan: A Qualitative Study

**DOI:** 10.1111/jjns.70086

**Published:** 2026-11-03

**Authors:** Sanae Fukui, Mari Matsuoka

**Affiliations:** ^1^ Wakayama Faculty of Nursing Tokyo Healthcare University Wakayama Japan; ^2^ School of Nursing, Faculty of Medicine Mie University Tsu Japan

**Keywords:** child, family nursing, home nursing, nurse–patient relations, qualitative research

## Abstract

**Aim:**

Children with medical complexity require ongoing medical and daily care at home, placing substantial demands on their families. In this study, we aimed to elucidate the experiences of home‐visiting nurses caring for children with medical complexity, focusing on how they build relationships with primary caregivers and children and deliver care within this relational context.

**Methods:**

This qualitative descriptive study included 22 home‐visiting nurses in Japan. Data were collected through semistructured interviews and analyzed using thematic analysis.

**Results:**

Four main themes were identified: “Understanding the mother and child within their everyday lives,” “Being there for the mother as a professional nurse,” “Developing reciprocal relationships within the mother–child–nurse triad,” and “Extending relational nursing care through supportive networks.” Nurses integrated the mothers' experiential knowledge, children's responses, and their own professional observations to negotiate individualized care. Their relational practice extended beyond the mother–child–nurse relationship to wider family and professional support networks. “Nurses' emotional engagement in relational nursing” was identified as a cross‐cutting process.

**Conclusions:**

Pediatric home nursing for children with medical complexity involves relational and negotiated practice in which nurses seek to understand and support mothers and children, develop reciprocal relationships, and extend relational care to wider support networks. The findings of this study highlight the importance of integrating parental experiential knowledge, children's responses, and professional assessment in negotiating individualized care, while maintaining a professional responsibility for children's safety and rights.

## 1 Introduction

Advances in neonatal and pediatric medicine have increased the number of children worldwide who require ongoing medical care while living in community settings (Falkson et al. 2017). In Japan, ~20,000 children require daily medical care (Children and Families Agency of Japan 2024). The well‐recognized concept Children with Medical Complexity (CMC) has been used internationally to describe children with complex health care needs who require substantial health care services (Cohen et al. 2011). Caring for CMC requires intensive daily caregiving and complex medical care, placing a substantial burden on families. Caregivers frequently experience physical, psychological, and social challenges, including sleep deprivation, depressive symptoms, and social isolation (Feeley et al. 2014; Bayer et al. 2021; Ali et al. 2025; Crettenden et al. 2018). Evidence‐based home‐visiting nursing and supportive relationships with home‐visiting nurses can enhance parental satisfaction, self‐efficacy, and confidence in caregiving (Kanda et al. 2022; Mas et al. 2019; Satherley et al. 2021); however, inadequate communication and a lack of shared understanding between families and healthcare professionals remain significant barriers to high‐quality, safe care (Crow et al. 2002; Jackson et al. 2001). Establishing trust and collaborative relationships with families is fundamental to home‐visiting nursing for CMC. Such relationships enhance family satisfaction, promote safe, high‐quality care, and support families in managing the ongoing demands of caregiving. Although previous studies have examined care satisfaction and parental self‐efficacy pertaining to nurse–family relationships, little is known about how home‐visiting nurses develop and sustain these reciprocal relationships in everyday practice. Greater insights into nurses' experiences and perceptions while building relationships with families may assist in strengthening nurse–family partnerships and improving home‐based care quality for CMC.

Mothers are often primary caregivers for CMC, assuming substantial responsibility for complex medical care and everyday family life (Ministry of Health, Labour and Welfare of Japan 2021; Satherley et al. 2021). In Japan, traditional gender roles can become more pronounced in families of children with disabilities, contributing to disproportionate and sustained caregiving burdens on mothers and affecting their employment and social activity participation (Kanai and Suda 2022; Haruki 2020). Such intensive caregiving responsibilities may affect the caregivers' health, their quality of life, and the well‐being of their extended family (Kaydok et al. 2020; Öztürk and Alemdar 2023). Establishing a trusting and collaborative relationship with mothers is fundamental to family‐centered pediatric home nursing care. We aimed to evaluate the experiences of home‐visiting nurses who provide care to CMC, focusing on how they build relationships with primary caregivers and children and deliver care within this relational context.

## 2 Methods

### 2.1 Study Design

We used qualitative inductive methods and thematic data analyses. Data were collected through semistructured interviews—transcribed verbatim—and data were included in the thematic analysis (Braun and Clarke 2006; Livingston et al. 2019; Paton et al. 2013).

### 2.2 Participants and Procedures

Participants were initially recruited (April 2018–December 2018) through convenience and snowball sampling (Meadows et al. 2001). During the initial recruitment phase (convenience sampling), the lead researcher provided a written study description (including the study description, consent form, consent withdrawal form, and mailing address set by the author) to five home nursing station managers that were active in pediatric homecare across Japan; four of these stations participated in the study. The administrators identified potential participants, informed them of the study, and advised them to contact the researchers directly in writing if they decided to participate in the study. In the next recruitment stage (snowball sampling), participants from the first stage were screened to include only pediatric home‐visiting nurses. Eligible nurses included those with experience in providing home nursing care to children aged < 6 years requiring medical care (with families receiving homecare for ≥ 1 year), ≥ 5 years of clinical experience in a medical facility, and ≥ 3 years of experience as a home‐visiting nurse (or a home healthcare agency administrator recommendation). Nurses who agreed to participate in the study were contacted by the researchers.

Overall, 22 home‐visiting nurses from 13 home healthcare agencies in seven rural areas of the country were willing to participate in the study. After confirming the attainment of informed consent, interviews were arranged. Each participant was interviewed separately. The first author conducted pilot interviews with two participants to test the interview questions and gain experience before study commencement. We rephrased the interview questions to ensure data quality and more in‐depth responses from participants based on the pilot interview results. The first author had prior experience in conducting qualitative interviews and underwent additional training through the present study's pilot interviews.

Data were collected through semistructured interviews. The first author traveled to the locations designated by the participants (often spare rooms within home‐visiting nurses' stations) to conduct the interviews. Each interview lasted for ~60 min, was recorded, and notes were taken on an Informed Consent recorder with the participants' consent. One interview was conducted with each participant. Home‐visiting nurses were encouraged to freely discuss their thoughts, perceptions, and experiences regarding the care provided to families with CMC requiring medical care. The interview guide had the following instructions: (1) please talk about the CMC you have been assigned to and their families and what you have experienced since you started working with them, (2) please talk about your thoughts, perceptions, and feelings from your interactions with the CMC and their mothers, and (3) please talk about how you felt about the changes over time since encountering the CMC and their mothers. Data saturation was determined at the 22nd participant interview, at which point no new codes emerged; therefore, no additional interviews were conducted.

The first author—a nurse and nursing researcher—conducted all the interviews, but had no prior acquaintance with the participants and first contacted them for this study through snowball sampling. The researchers reflected on how their professional backgrounds might influence the interpretation of the participants' accounts and discussed these potential influences with the co‐authors during the analysis process.

### 2.3 Data Analysis

All interviews were transcribed verbatim and analyzed. We used Braun and Clarke's method (Braun and Clarke 2006) of thematic analysis, which identifies, analyzes, and reports patterns of meaning in data in each phase and is adaptable to various theoretical and epistemological approaches. The analysis process began with a verbatim transcription of the interviews recorded on the Informed Consent recorder by the first author (Phase 2). During the coding process, the data were coded based on similarities, relationships, and differences. The co‐researchers discussed the coding until reaching a consensus, related codes were organized into code clusters, and potential themes and their overarching domains were identified collaboratively (Phase 3). In Phase 4, we examined the relevant codes, and each code was compared among co‐authors throughout the entire process. When consolidating the themes, all classifications were reviewed and defined until a final agreement was reached among the co‐authors (Phase 5). In Phase 6, patterns were ultimately identified, and the overall narrative was examined.

By employing Braun and Clarke's method of analysis, themes and subthemes were generated. The relationships among the themes were examined to identify patterns in how nurses' relational practices developed and extended across their interactions with the mothers, children, and others involved in care. The narratives were reviewed individually for each participant, and the overall thematic flow was identified accordingly. Themes were refined through repeated examination of code coherence within each theme and the relevance of each theme in relation to the entire dataset. The representation of themes and thematic maps were discussed between both authors until mutual agreement was reached. Feedback on the results was sought from one study participant experienced in qualitative research. Theme and subtheme generation were explained, and an agreement on the thematic map was reached. This process was conducted as a member check by the participant. All interviews were transcribed and analyzed in Japanese.

### 2.4 Ethical Considerations

Kyoto University Graduate School and Faculty of Medicine, Ethics Committee approved the study (ref. R1330). All participants were aware of the purpose, methods, and importance of the study, and each participant signed a written informed consent form prior to registration. All interview details (including audio recordings) were kept confidential. Although children and family members were not research participants, child safety was considered throughout the study. Any concerns regarding child safety, abuse, neglect, or inappropriate care could be reported to the manager of the home‐visiting nursing station. Participants were informed about confidentiality but were not specifically informed of this reporting procedure during consent.

### 2.5 Rigor

The proposed criteria (Guba and Lincoln 1982) ensured that the entire process was conducted with rigor. First, the data collection instrument and interview guide were pre‐tested twice and modified to ensure that the intent of the questions was clear. To ensure confirmability throughout the study, the researcher carefully documented the collected data and its interpretation by checking the methods employed and inter‐researcher beliefs. Member checking was commissioned with interviewees with research experience to confirm data reliability. All stages, from research protocol development to final report preparation, were reviewed until the researchers reached a consensus. Transferability was ensured: interviews were carefully transcribed, and the researcher's subjective ideas were quoted and described verbatim, excluding the participants' experiences. This study followed the Consolidated Criteria for Reporting Qualitative Research (Tong et al. 2007).

## 3 Results

### 3.1 Study Participants

Twenty‐two home‐visiting nurses were interviewed. The study participants had a mean of 22 years (range 10–36 years) of nursing experience (Table 1). Some participants held additional qualifications, including Certified Nurse and childcare worker qualifications, and participants varied in their experience of pediatric nursing. Account comparisons did not reveal clearly distinguishable patterns of relationship‐building according to these professional characteristics. The children receiving care from the participating nurses were aged 2–5 years and had received homecare for 1.5–4.0 years. Their medical care needs included tracheostomy, respiratory management, enteral tube feeding, oxygen therapy, enemas, and urinary drainage. Most were unable to move independently and required extensive daily care assistance. The average interview time was 45 min (range 30–120 min).

**TABLE 1:** Demographic characteristics of the sample (*N* = 22).

Gender				
	Women			21
	Men			1
Age, years		Mean (range)		46.1 (39–56)
Title				
	RN			22
	Certified nurse (specialized: home‐visiting nursing and pediatric emergency nursing)			4 (18%)
	Early childhood educator			2 (9%)
Years of work experience				
	Years of nursing experience		Year: Mean (range)	22.8 (10–36)
		General nursing (without pediatrics)	Year: Mean (SD)	13.9 (±6.5)
		Pediatric nursing		11.7 (±4.3)
		Home‐visiting nursing		7.5 (±4.3)
		Pediatric home‐visiting nursing		7.5 (±4.3)

Abbreviations: RN, registered nurse; SD, standard deviation.

The analysis identified four themes describing home‐visiting nurses' experiences of building relationships with mothers and CMC and providing care within these relationships: (1) Understanding the mother and child within their everyday lives, (2) Being there for the mother as a professional nurse, (3) Developing reciprocal relationships within the mother–child–nurse triad, and (4) Extending relational nursing care through supportive networks. The nurses' emotional engagement in relational nursing was identified as a cross‐cutting process that intersects with the relational practices represented in these themes. The themes, subthemes, and cross‐cutting processes are presented in Table 2.

**TABLE 2:** Themes and subthemes.

Themes	Subthemes
Theme 1	Sensing the mother's thoughts and feelings
Understanding the mother and child within their everyday lives	Understanding the child's vulnerabilities and abilities
Theme 2 Being there for the mother as a professional nurse	Supporting maternal identity under the burden of medical and childcare responsibilities Providing emotional support through professional presence Remaining present despite difficulties
Theme 3 Developing reciprocal relationships within the mother–child–nurse triad	Sharing observations and knowledge about the child's development Developing mutually acceptable care with the child Recognizing interactions within the mother–child–nurse triad
Theme 4 Extending relational nursing care through supportive networks	Connecting mothers with other mothers Supporting children's everyday lives with siblings Collaborating with professionals around shared goals
Cross‐cutting process: Nurses' emotional engagement in relational nursing	Experiencing mothers' difficulties as one's own emotional concern
	Sharing mothers' joy in their children's development

### 3.2 Theme 1

Understanding the mother and child within their everyday lives.

Across participants' accounts, understanding mothers and children within the context of their everyday lives was central to home‐visiting nursing practice. This theme comprised two subthemes: (1) Sensing the mother's thoughts and feelings, and (2) Understanding the child's vulnerabilities and abilities. Nurses sought to understand not only the child's medical and developmental characteristics but also the mother's experiences and knowledge gained through everyday caregiving. This understanding formed a basis for tailoring nursing care to the individual child and family.

#### 3.2.1 Sensing the Mother's Thoughts and Feelings

Nurses sought to understand mothers' thoughts, feelings, and experiences related to caring for their children. They perceived mothers' feelings of guilt, strong beliefs about child‐rearing, and difficulties in expressing their concerns.

At the early stage of home visits, nurses sought to understand the emotional struggles that mothers experienced in caring for their children. For example, one nurse perceived that a mother whose child had recently been discharged from a medical facility felt a sense of guilt toward her child, who required ongoing medical care. The nurse also perceived that the mother believed she should manage all aspects of her child's medical care independently.

“Yes, I think she has become somewhat one‐sided in her thinking. It seems she carries a sense of self‐blame for having given birth to a small, fragile child, and feels that she must manage everything on her own.” (Nurse 15)

Nurses also recognized that mothers had strong beliefs about how they wanted to raise their children. For example, one nurse described a mother who experienced considerable stress when her own mother repeatedly offered to take care of the child.

“The mother was told many times by her own mother, ‘I will take care of your child,’ and every time this was said, she felt a lot of stress. As the mother wants to do things in her own way, she does not want to have the child taken away from her.” (Nurse 22)

However, nurses found that mothers' worries and struggles were not always readily accessible. Since nurses were entering the family's private home, they were cautious about asking questions too directly.

“Since the nurse is the one entering the home, it is not possible to be too pushy when asking questions. Gradually, the mother started to express her needs to me, but I still want to understand the feelings behind those needs. It is quite difficult, though. Just when I feel like I am getting closer, it seems like she pulls away again.” (Nurse 11)

These accounts illustrate that understanding mothers' experiences is built through repeated encounters and attentive engagement rather than through a single assessment. Such understanding enabled nurses to consider mothers' perspectives when determining how to provide care within the family's everyday life.

#### 3.2.2 Understanding the Child's Vulnerabilities and Abilities

Nurses assessed the children's physical vulnerabilities and abilities from the beginning of home visits. They sought to understand the children's physical characteristics, developmental potential, and responses to their surroundings.

Nurses described children whose physical conditions could deteriorate rapidly and recognized that unfamiliar people and care could cause psychological distress that may also affect their physical condition.

“The child had conditions like bronchomalacia, so when they cried, their oxygen saturation would drop significantly. The mother was really on edge because of that, and the child would cry more when I visited. It was a really tough time for each of us, every time I went.” (Nurse 5)

Nurses also attended to the children's developmental abilities by observing their responses during everyday interactions.

“When he sees things or hears music playing like that, he reaches out toward it. When his siblings are, well, making a lot of noise, he also smiles and looks happy. His reactions to those are definitely getting better.” (Nurse 3)

Nurses recognized that mothers often possessed a deeper understanding of their children because of their continuous involvement in everyday care. At the same time, nurses used their professional observations to complement this experiential knowledge.

“I think there are a lot of things that only the mother would truly understand. Even though I can understand some, I believe the mother understands much more. I always feel like I need to fill in that gap somehow.” (Nurse 1)

Conversely, nurses sometimes identified physical or developmental characteristics that mothers had not recognized, illustrating the complementary nature of the mothers' experiential knowledge and nurses' professional assessment.

“Then the child started imitating her older sister, and I thought, ‘Oh, she's copying her!’ But the mother did not really pick up on it.” (Nurse 15)

Thus, the nurses' understanding of the child involved both valuing mothers' experiential knowledge and contributing professional observations that could complement or extend that knowledge. Hence, nurses assessed the child's condition and development within the context of everyday family life rather than relying solely on clinical observations.

### 3.3 Theme 2

Being there for the mother as a professional nurse.

Across the interviews, “being there” for mothers emerged as an important aspect of professional nursing practice. This theme comprised three subthemes: (1) Supporting maternal identity under the burden of medical and childcare responsibilities, (2) Providing emotional support through professional presence, and (3) Remaining present despite difficulties. Rather than simply taking over caregiving tasks, nurses sought to support mothers while respecting their identities, values, and ways of caring for their children. Being present therefore involved an intentional nursing practice of providing support while maintaining the mother's role and agency in caring for her child.

#### 3.3.1 Supporting Maternal Identity Under the Burden of Medical and Childcare Responsibilities

Nurses described supporting mothers while respecting their individual ways of caring for their children. They sought to adapt their support to each mother's pace and preferences rather than simply taking over difficult aspects of childcare.

“Well, I think it is really important to go at the mother's own pace, and when we talk about easing her burden, it does not mean just taking over all the difficult parts of childcare. What really matters is supporting her in a way that allows her to do things how she wants to.” (Nurse 22)

Nurses therefore sought to support mothers without undermining their role as the primary caregivers, maintaining their active involvement in their children's care. Their practice involved negotiating support around the mother's preferences and capabilities rather than substituting professional care for maternal caregiving.

#### 3.3.2 Providing Emotional Support Through Professional Presence

Nurses described their physical and psychological presence as a source of emotional support for mothers. This was particularly evident during demanding periods such as the transition from hospital to home.

“I think the transition period—when the child moves away from the hospital and shifts to home visits—can be pretty tough for the mother. So, in those times, even though we might only visit once in the morning and once in the evening, just being there can make a big difference.” (Nurse 1)

The nurse's role therefore extended beyond direct care to include being available as a professional whom mothers could consult and rely on. Through this professional presence, nurses provided mothers with a continued source of support during periods of substantial caregiving demands.

#### 3.3.3 Remaining Present Despite Difficulties

Some nurses described remaining present despite difficulties in establishing relationships with families. For example, one nurse described experiencing rejection from a family but nevertheless choosing to remain present.

“The family did not expect [medical assistance], so I did not really do anything. Just standing there and watching—that was actually pretty hard. Still, even without doing anything, being there as a nurse in that moment felt meaningful.” (Nurse 5)

These accounts illustrate how being present could represent an intentional professional practice through which nurses maintain their relationship with the family even when the immediate intervention was difficult or not welcomed.

### 3.4 Theme 3

Developing reciprocal relationships within the mother–child–nurse triad.

Across participants' accounts, relationships with mothers and children were described through ongoing interactions. This theme comprised three subthemes: (1) Sharing observations and knowledge about the child's development, (2) Developing mutually acceptable care with the child, and (3) Recognizing interactions within the mother–child–nurse triad. Nurses described reciprocal interactions with mothers and children through repeated home visits. Although the mother–child–nurse relationship was explicitly articulated as a three‐way interaction in some accounts, the broader data showed interconnected interactions between nurses and mothers and between nurses and children. Nurses responded to these interconnected relationships and incorporated them into how they understood and provided care.

#### 3.4.1 Sharing Observations and Knowledge About the Child's Development

Some nurses described sharing observations and knowledge about children's development with mothers. Nurses communicated subtle changes they observed, while mothers contributed observations derived from everyday family life.

“Saying something like, ‘Looks like the corners of his mouth are turned up today,’ would make the mother brighten up and say, ‘Yes, that is right. Last night, actually…’ and the conversation would flow. So, I think keeping those little moments going through the child is really important.” (Nurse 1)

Through these exchanges, nurses and mothers developed a shared understanding of the child by bringing together professional observations and mothers' experiential knowledge. This reciprocal sharing enabled nurses to incorporate the mother's knowledge of the child's everyday behavior into their nursing assessment while contributing observations from a professional perspective.

#### 3.4.2 Developing Mutually Acceptable Care With the Child

Nurses described adjusting their care in response to the children's subtle physical and behavioral responses. They sought to avoid discomfort and identify ways of providing care that children could accept.

“The child seemed to understand that the nurse was someone who listened and would stop at a comfortable point and gradually began to smile during care.” (Nurse 2)

The child's responses therefore became an important source of information for nurses' ongoing assessment and adjustment of care. Rather than determining care solely from the nurse's perspective, nurses treated children's behavioral and physical responses as meaningful expressions that informed how care was provided. Through repeated interactions, children came to recognize nurses as people who responded to their signals, contributing to a mutually acceptable way of interacting.

#### 3.4.3 Recognizing Interactions Within the Mother–Child–Nurse Triad

Some nurses explicitly recognized interactions involving the mother, child, and nurse as a three‐way relationship. For example, one nurse described how a mother's new understanding of her child's behavior led to changes in the home environment and subsequently to changes in the child's engagement.

“The mother said something like, ‘When the TV is on, I realized—oh, maybe he cannot concentrate,’ and I was really happy to hear that. After that, she started turning off the TV during childcare, and the child started showing more interest and trying really hard to engage in what we were doing.” (Nurse 13)

Here, the nurse's observations contributed to the mother's understanding of the child, which influenced the mother's interaction with the child and was followed by changes in the child's responses. This account illustrates how nursing practice could be situated within an interconnected mother–child–nurse relationship, in which interactions among the three shaped the care provided.

### 3.5 Theme 4

Extending relational nursing care through supportive networks.

Across the participants' accounts, relational nursing care extended beyond the immediate mother–child–nurse relationship to wider support networks. This theme comprised three subthemes: (1) Connecting mothers with other mothers, (2) Supporting children's everyday lives with siblings, and (3) Collaborating with professionals around shared goals.

Nurses described extending their support to include peer connections, siblings, and healthcare professionals. These accounts indicate that nurses understood the child's care not as an isolated clinical task but as part of the child's everyday family and social environment.

#### 3.5.1 Connecting Mothers With Other Mothers

Some nurses described facilitating connections among mothers caring for CMC. They recognized that mothers could provide one another with practical knowledge and emotional support that professionals could not always offer.

“I realized that the mothers talking to each other is more helpful than nurses telling them all sorts of things. Even with suctioning … an experienced mother would share, ‘This is how we did it at our house.’ Having another mother you can consult with—I thought that was really great.” (Nurse 12)

Rather than positioning themselves as the sole source of knowledge, nurses recognized the value of mothers' experiential knowledge and supported opportunities for mothers to learn from one another. Hence, nurses facilitated supportive connections through which the mothers' lived experiences could become a resource for sustained homecare.

#### 3.5.2 Supporting Children's Everyday Lives With Siblings

Nurses also described attending to children's relationships with siblings and incorporating siblings into everyday activities.

“When I go to visit, it is like I play with the big sister too. I will say, ‘Hey sis, give me a hand—this is tough to do alone,’ and she will come over saying, ‘Yeah, I might be better at this,’ and help wash the kids' feet or something, doing things together.” (Nurse 8)

Through such interactions, nurses support not only the children's medical care but also their participation in everyday family life. Nursing care thus extended to preserving children's opportunities to participate in family relationships and ordinary activities with their siblings.

#### 3.5.3 Collaborating With Professionals Around Shared Goals

Nurses described collaborating with other professionals and mothers around shared goals for the child. They considered the children's potential and discussed achievable goals together with their mothers and other professionals.

“Ultimately, the goal is for her to be able to sit independently. Although standing while supporting her whole body remains difficult, we have discussed with the physical therapist and mother that it would be ideal to reach that stage. We are planning to proceed step by step.” (Nurse 4)

These accounts illustrate how nurses drew on their relationships with mothers and children when coordinating care with other professionals. Rather than coordinating services solely around medical needs, nurses worked with mothers and professionals to establish shared goals that reflected the child's developmental potential and everyday life.

### 3.6 Cross‐Cutting Process: Nurses' Emotional Engagement in Relational Nursing

Emotional engagement was also evident in the nurses' accounts of their relationships with mothers and children. Nurses described sharing the mothers' joy in their children's development and, sometimes, experiencing the mothers' difficulties as their own emotional concern. These emotional responses were intertwined with the nurses' ongoing relational engagement.

#### 3.6.1 Experiencing the Mothers' Difficulties as One's Own Emotional Concern

Some nurses described experiencing emotional distress in response to the mothers' suffering. For example, one nurse described feeling a mother's pain as she observed the mother's persistent self‐blame and intense sense of responsibility for her child's well‐being.

“The mother herself was constantly scrutinizing every little thing and blaming herself. The mother had this feeling that if she stopped, her child would stop too. So, watching the mother like that, I started feeling the pain too. I thought, ‘Oh, that must be painful,’ and I felt it too.” (Nurse 12)

#### 3.6.2 Sharing Mothers' Joy in Their Children's Development

Nurses also described experiencing joy together with mothers when they observed positive changes in their children's responses or development.

“When a food got a good reaction from the child's taste buds, the mom showed me how the child was eating it. I think she wanted me to see her child's reaction; I was really happy at that moment. I wanted to share that joy together with the mom.” (Nurse 8)

Thus, the nurses' emotional experiences were intertwined with the relationships they developed with mothers and children. Emotional engagement accompanied sustained relational involvement, through which nurses shared in both the difficulties and positive changes experienced by mothers and children.

## 4 Discussion

We found that pediatric home nursing for CMC involves building relationships with mothers and children and negotiating individualized care by integrating mothers' experiential knowledge, children's responses, and nurses' professional observations. The themes were interconnected rather than sequential, representing overlapping aspects of relationship‐building in home‐visiting nursing practice. Previous studies have primarily described caregiver–nurse relationships in homecare (Nageswaran and Golden 2018) or dyadic nurse–parent and nurse–child partnerships (Barratt et al. 2024). Contrastingly, our findings illustrate how these relationships are interconnected within a mother–child–nurse triad and extend to wider family and professional support networks.

Being there for the mother as a professional nurse appeared to provide an important starting point for this relational process. Nurses sought to understand mothers' values, concerns, and difficulties while respecting their ways of caring for their children. Parents of CMC assume substantial responsibility for their children's care at home and become experienced care providers (LeGrow et al. 2022b). Previous studies have similarly emphasized communication and trust in developing caregiver–nurse relationships in homecare (Nageswaran and Golden 2018) and meaningful connections between homecare nurses and children and their families (Mekhuri et al. 2025). This approach is consistent with partnership nursing, which emphasizes mutual trust and respect, communication, negotiation, and parental participation (Barratt et al. 2024). In our study, understanding mothers enabled nurses to interpret the child's condition within family life and adapt care while maintaining the mother's active role. Thus, professional presence provided a foundation for trust and subsequent reciprocal relationships.

As nurses became familiar with mothers and children through repeated visits, these relationships increasingly involved reciprocal observation processes and care negotiation. Similarly, previous research pertaining to CMC found that mother–nurse decision making was relational, dynamic, and collaborative (LeGrow et al. 2022a). Nurses in our study recognized mothers' experiential knowledge of their children's everyday lives while contributing professional observations of the children's physical condition and development. Studies of decision‐making for CMC emphasize the importance of parental knowledge and experience alongside professional expertise (Lin et al. 2020; Buchanan et al. 2022). Community health service research further highlights collaboration, continuity, communication, and parental knowledge in shared decision‐making (Jacobs et al. 2023). Nurses additionally attended to children's subtle behavioral and physical responses and adjusted care accordingly. Thus, the mothers' experiential knowledge, children's responses, and nurses' professional observations were integrated to negotiate individualized care.

The reciprocal relationships identified in this study can be understood in relation to Randall's pragmatic children's nursing theory, which conceptualizes children's nursing within a triadic relationship among the child, carer, and nurse (Randall 2016). Collectively, these findings suggest that relationships with mothers and children were interconnected, suggesting that nurses' interactions with each could be understood within a broader mother–child–nurse relational context. Previous research in pediatric nursing has similarly described reciprocal collaboration among children, parents, and nurses (Sundal and Vatne 2020). However, partnership nursing has more often been described through separate nurse–parent and nurse–child dyads, with limited evidence of how triadic partnerships are enacted in practice (Barratt et al. 2024). Children with long‐term conditions value being recognized as individuals within such relationships and having opportunities to participate in decisions about their care (Barratt et al. 2023). In our study, nurses attended to children's behavioral and physical responses while integrating the mothers' experiential knowledge and their own professional observations. Thus, the findings illustrate how a mother–child–nurse relational context may inform reciprocal understanding and negotiated care in everyday pediatric home nursing.

Importantly, these relational practices were embedded within the nurses' professional assessment and clinical decision‐making. In the home setting, nurses interpreted children's physical and behavioral responses within everyday family life while balancing the parents' knowledge and preferences with professional responsibility for the child's care. This is consistent with family‐centered pediatric care, which conceptualizes families and healthcare professionals as partners in care and decision‐making (Kuo et al. 2012). Studies of home‐based pediatric care further emphasize the nurses' abilities to recognize changes in children's conditions, adapt care to individual needs, and negotiate safety and responsibility with families (Aasen, Johannessen, Knutsen, and Werner 2022; Aasen, Johannessen, Ingrid Ruud Knutsen, and Werner 2022). For CMC, professional knowledge, communication with parents, continuity, and coordination across services are also important components of care (Haspels et al. 2025). In Japan, parents of CMC have identified insufficient availability of pediatric‐trained home‐visiting nurses and weak interagency coordination as challenges to homecare (Yamaguchi and Suzuki 2026). Thus, the relationships identified in our study provide a context for professional judgment, adaptation of care, and responsibility for the child within family life.

The relational process also extended beyond the mother–child–nurse relationship to extended family, peer, and professional networks. Nurses connected mothers with other parents, supported children's everyday relationships with siblings, and collaborated with professionals around shared goals. Peer support can provide caregivers of CMC with informational and social support and opportunities to share resources (Kelly et al. 2022). Caregiving for CMC also affects relationships and functioning across the whole family, highlighting the importance of support that extends beyond the primary caregiver (de Groot et al. 2026). Home healthcare nurses caring for these children collaborate with multiple providers, although challenges in communication, information sharing, and clinical problem‐solving remain (Nageswaran and Golden 2016). More broadly, care coordination and integrated care can facilitate navigation across services and communication among care teams (Van Orne 2022; Zurynski et al. 2025). In Japan, parents have similarly identified peer support and information sharing as important resources, while insufficient interagency coordination remains a challenge (Yamaguchi and Suzuki 2026). Thus, home‐visiting nurses may help connect children's and families' everyday lives with broader social and professional support networks.

Emotional engagement was also intertwined with these relational practices. Nurses shared the mothers' joy in their children's development and, at times, experienced the mothers' difficulties as their own emotional concern. Close and continuing relationships with children and families can foster emotional involvement, while also creating challenges in maintaining appropriate professional boundaries (Fegran and Helseth 2009; Barratt et al. 2024). Similar challenges have been reported specifically in homecare for CMC, where caregivers and nurses described difficulties navigating professional, personal, and emotional boundaries (Nageswaran and Golden 2018). Emotional engagement in nursing also involves the regulation of emotions within caring relationships (Meneghini et al. 2024), highlighting the need for nurses to remain aware of how emotional involvement shapes their professional practice. Thus, emotional engagement in our study may be understood as part of relational nursing that requires reflection and careful boundary management, rather than as evidence that greater emotional involvement necessarily leads to better care.

Some limitations should be considered. Participants were experienced home‐visiting nurses recruited through snowball sampling, which may have introduced selection bias and limited the diversity of perspectives. Only one participant was male, and all primary caregivers represented in participants' accounts were mothers, limiting exploration of how gender and different caregiving arrangements may shape relational practice. The findings reflect nurses' perspectives and may also have been influenced by differences in clinical backgrounds, children's conditions, and regional contexts. Although the first author had no prior acquaintance with participants, the first author's professional background as a nurse and nursing researcher may have influenced the research process and interpretation. Some linguistic and cultural nuances in participants' Japanese expressions may not have been fully conveyed in English. Child safeguarding and children's rights were not specifically investigated, and procedures for reporting safety concerns were not explicitly explained during consent. Future research should include children, diverse family caregivers, and examine how their perspectives, gender, and caregiving arrangements shape relationships among children, families, and home‐visiting nurses.

## 5 Conclusions

This study suggests that pediatric home nursing for CMC involves a relational process in which nurses seek to understand and support mothers and children, develop reciprocal relationships within the mother–child–nurse triad, and extend relational care to wider family and professional support networks. Nurses integrated mothers' experiential knowledge, children's responses, and their own professional observations to negotiate individualized care. These findings highlight the importance of relational and professional competencies in pediatric home nursing, including attention to children's responses, collaboration with families, and coordination with other professionals. Future research should examine these relational processes from the perspectives of children and diverse family caregivers.

## Author Contributions

Sanae Fukui contributed to the conception and design of this study; Sanae Fukui and Mari Matsuoka performed the analysis; Sanae Fukui drafted the manuscript; and Mari Matsuoka critically reviewed the manuscript and supervised the whole study process. All authors read and approved the final manuscript.

## Funding

The study was funded by the Yuumi Memorial Foundation for Home Health Care.

## Ethics Statement

Kyoto University Graduate School and Faculty of Medicine, Ethics Committee approved the study (ref. R1330).

## Consent

All participants provided informed consent.

## Conflicts of Interest

The authors declare no conflicts of interest.

## Data Availability

Due to privacy and ethical considerations, this data is not publicly available.
